# Alcohol Dehydrogenase 1B Suppresses β-Amyloid-Induced Neuron Apoptosis

**DOI:** 10.3389/fnagi.2019.00135

**Published:** 2019-06-05

**Authors:** Yaqi Wang, Yi Zhang, Xiaomin Zhang, Tingting Yang, Chengeng Liu, Peichang Wang

**Affiliations:** Clinical Laboratory of Xuanwu Hospital, Capital Medical University, Beijing, China

**Keywords:** Alzheimer’s disease, alcohol dehydrogenase 1B, β-amyloid, p75NTR, apoptosis

## Abstract

β-amyloid (Aβ) deposition, neurofibrillary tangles induced by phosphorylation of tau protein, and neuronal apoptosis are pathological hallmarks of Alzheimer’s disease (AD). The dementia rate in alcoholic abusers were found to be higher than in control people. The present study explored the potential roles of alcohol dehydrogenase 1B (ADH1B) in AD pathology by determining the ADH1B levels in AD patient sera, in the hippocampus of APP/PS-1 AD model mice, and in an AD model cell line treated with Aβ1-42. The results show that ADH1B levels decreased significantly both in the serum of AD patients and in the hippocampus of APP/PS-1 AD model mice. In addition, the apoptotic rate was reduced and viability was significantly increased in AD model cells transfected with ADH1B overexpression vector. The levels of the p75 neurotrophin receptor (p75NTR), an Aβ1-42 receptor, were down-regulated in the ADH1B overexpressing AD model cell and up-regulated in cells transfected with the shRNA vector of ADH1B. Protein levels of cleaved caspase-3 and Bax decreased significantly, whereas Bcl-2 levels increased in cells overexpressing ADH1B. The opposite trend was observed for cleaved caspase-3, Bax, and Bcl-2 levels in cells transfected with the shRNA vector of ADH1B. The levels of reactive oxygen species (ROS) were found to be reduced in ADH1B overexpressing cells and increased when cells were transfected with the shRNA vector of ADH1B. These results indicate that ADH1B might be important in the prevention of AD, especially for abusers of alcohol, and a potential new target of AD treatment.

## Introduction

Alzheimer’s disease, a progressive neurodegenerative disorder, is characterized by cognitive impairment and behavioral changes (Kobayashi and Chen, 2005; Lawrence et al., 2017). It is considered to be a result of Aβ accumulation, neurofibrillary tangles (NFT) induced by the phosphorylation of tau protein, and neuron apoptosis in the brain (Copani et al., 2002; Sealey et al., 2017). Previous studies demonstrated that Aβ1-42 promotes autophagy and induces apoptosis of neurons, which could underlie its up-regulation of stress levels and neurotoxicity (Bozyczko-Coyne et al., 2001; Xue et al., 2014; Hung et al., 2015). The metabolic rate of Aβ1-42, the product of the amyloid precursor protein (APP), is dependent on proteins such as β-site APP cleaving enzyme 1 (BACE 1), IDE, and p75NTR (Hirata-Fukae et al., 2008; Hampel and Shen, 2009; Saadipour et al., 2013; Ito et al., 2016; Kurochkin et al., 2018).

Early epidemiological studies showed that dementia frequency in alcoholic abusers is higher than in control people (Kato, 1991; Davis, 1993; Topiwala et al., 2017). ROS-dependent lipid peroxidation results in toxic aldehydes such as MDA, acetaldehyde, and 4-HNE, which enhance oxidative stress levels in age-related diseases (Ansari et al., 2011; Yonny et al., 2015). Acetaldehyde, the substrate of acetaldehyde dehydrogenase (ALDH) and the main metabolite of ethanol, can mediate cognitive dysfunction and brain tissue damage induced by the chronic excessive consumption of alcohol (Yan et al., 2016). In addition, ALDH2 decreases the accumulation of the lipid peroxidation product 4-hydroxynonenal (HNE) in the AD brain and could be associated with AD pathology (Bai and Mei, 2011; D’Souza et al., 2015).

Alcohol dehydrogenases (ADHs) are another group of important enzymes in the generation of acetaldehyde. There are many isoforms of ADH, which are classified into different classes based on substrate specificity and catalytic properties: ADH1–ADH6. ADH1 plays the most crucial role mainly during alcohol metabolism (Li et al., 2017). Human ADH1 is the only class consisting of three isoenzymes, namely ADH1A, ADH1B, and ADH1C (Duester et al., 1999). Interestingly, the down-regulation of ADH1B was observed in the serum of AD patients using chip assays and mass spectrometric analysis in our previous work (not shown). The hypothesis that ADH1B could be involved in the pathology of AD promptly attracted our interest. In this study, APP/PS-1 AD model mice at different ages and the serum of AD patients were used to observe the differences in ADH1B expression in AD. SH-SY5Y, a human neuroblastoma cell line, was also used to prepare an AD model cell with Aβ1-42 incubation (Arai et al., 2016; Oguchi et al., 2017; Wu et al., 2017). The attenuation of apoptosis by ADH1B and its underlying mechanism, including Aβ1-42 production-associated proteins, Aβ1-42 receptor, apoptosis-related proteins, and oxidative stress levels, were analyzed using SH-SY5Y cells transfected with an ADH1B-containing vector.

## Materials and Methods

### Human Serum Samples

The design of this study was approved by the ethics committee of Xuanwu Hospital of Capital Medical University, and all patients or their legally authorized representatives provided individual informed consent. A total of 94 subjects were enrolled, including 37 patients with dementia of the Alzheimer type (DAT) (mean age = 70 years, ranging from 65 to 87), 30 patients with PD (mean age = 70.5 years, ranging from 65 to 82), and 27 age-matched healthy controls (mean age = 71 years, ranging from 60 to 83). Precise diagnosis was made by doctors of the Neurology Department of Xuanwu Hospital of Capital Medical University. None of the patients had other malignancies or active pulmonary disease (Table 1). Approximately 2 mL of serum was obtained and stored in liquid nitrogen until use (Zhang et al., 2016). The study was conducted in accordance with the guidelines of the Declaration of Helsinki.

**TABLE 1:** Demographic characteristics of the enrolled populations.

**Group**	**Age(y)**			**Gender(N)**		
	**Median (range)**	**Mean ± SEM**	***p*-Value**	**Female N(%)**	**Male N(%)**	***p*-Value**
HC *n* = 27	71(60–83)	71.3 ± 1.31	–	15(55.5)	12(45.5)	–
AD *n* = 37	70(65–87)	72.5 ± 1.12	HC vs. AD^a^n.s.	20(54.0)	17(46)	HC vs. PD^b^n.s.
PD *n* = 30	70.5(65–82)	71.9 ± 1.24	HC vs. PD^a^n.s.	15(50.0)	15(50)	HC vs. PD^b^n.s.

*HC, healthy controls, AD, Alzheimer’s disease, PD, Parkinson’s disease, y, years; n.s., not significant (*p* > 0.05). ^a^Mann–Whitney *U*-test. ^b^Chi-square test.*

### Animal Model

APPswe/PS1dE9 (Jackson Laboratory, Stock No. 004462, *n* = 30, male) and Prnp-SNCA^*^A53T (Jackson Laboratory, Stock No. 006823, *n* = 24, male) mice were purchased from Nanjing Biomedical Research Institute of Nanjing University. All animal experiments conformed to the National Institutes of Health guidelines. All animal procedures were approved by the ethics committee of Xuanwu Hospital of Capital Medical University. Model mice were kept with accessible water and feed under a 12 h light-dark cycle (Billings et al., 2005). Mice were separated into three groups: 4-month (AD, *n* = 10; PD, *n* = 8), 10-month (AD, *n* = 10; PD, *n* = 8), and 18-month (AD, *n* = 10; PD, *n* = 8) groups. C57BL/6J mice of corresponding ages formed the wild type group (WT, *n* = 8 per group).

### Lentivirus Transfection

We established ADH1B-overexpressing and shRNA-ADH1B SH-SY5Y cell lines using transduction of lentiviral vectors. ADH1B-overexpressing (NM_000668) and shRNA-ADH1B (TGACACC ATGATGGCTTCCCTGTTA) primers were synthesized. Control vectors (lentiviral-ADH1B corresponding to the ADH1B-overexpressing group and lentiviral-scramble corresponding to the shRNA-group) were used for comparison (Hanbio, Shanghai, China). SH-SY5Y cells were seeded onto six-well plates and transfected with these lentiviral vectors. Overexpression and interference effects were determined using western blotting after 48 h.

### Cell Culture and Treatment

The SH-SY5Y cell line was obtained from China Infrastructure of Cell Lines. Cells were maintained in DMEM:F12 medium using 10% fetal bovine serum and 1% penicillin–streptomycin at 37°C and 5% CO_2_ with humidified atmosphere. Cells were separated into four groups (shRNA con, shRNA ADH1B, overexpressing con, and overexpressing ADH1B) and transfected with the lentiviral vectors mentioned above. Next, cells were treated with 10 μM Aβ1-42 for 12 h (Bae et al., 2014). Cells were then harvested and prepared for the following tests.

### Preparation of Aβ1-42

Synthetic Aβ1-42 purchased from Abcam (United States, ab120301) was dissolved in 1,1,1,3,3,3–hexafluoro-2-propanol (HFIP, Sigma), incubated at room temperature for 1 h, gently mixed, and sonicated for 10 min. The solution was dried using nitrogen gas. The pellet was resuspended in 100% DMSO and incubated for 12 min at room temperature according to the manufacturer’s instructions (Olsen and Sheng, 2012). This Aβ1-42 stock solution was aliquoted, stored at −80°C, and equilibrated for 1 h at room temperature before use. The stock solution was diluted to a final concentration of 10 μM in DMSO.

### Enzyme-Linked Immunosorbent Assay (ELISA)

Whole blood was collected and kept at room temperature for approximately 30 min. After centrifugation at 2,000 × *g* for 30 min in a refrigerated centrifuge, supernatants were collected into microcentrifuge tubes and stored at −80°C until use. Serum ADH1B levels were determined using ELISA (Cloud-Clone) following the manufacturer’s protocol. Absorbance was measured at 450 nm using a microplate reader (Bio-Rad, Hercules, CA, United States).

### Western Blot Analysis

Hippocampus tissues were removed and homogenized in neuronal Protein Extraction Reagent (Thermo Fisher Scientific, 87792) containing a cocktail of protease and phosphatase inhibitors (Thermo Fisher Scientific, 87786). Protein concentrations were determined using the BCA protein assay (Thermo Fisher Scientific, 23227). Equal amounts of total protein were separated in 12% SDS–PAGE gels and then transferred to nitrocellulose (NC) membranes (Solarbio, Beijing, China). The primary antibodies were: rabbit anti-ADH1B (Biorbyt, 1:800), rabbit anti-BACE 1 (Abcam, 1:1000), rabbit anti-IDE (Abcam, 1:1000), rabbit anti-p75NTR (Cell Signaling Technology, 1:1000), rabbit anti-cleaved caspase-3 (Abcam, 1:1000), rabbit anti-Bcl-2 (Abcam, 1:1000), and rabbit anti-Bax (Abcam, 1:1000). Image Lab (Bio-Rad) was utilized for protein signal densitometry. Detection of proteins from pretreated SH-SY5Y cells using western blotting were performed as previously described (Zhang et al., 2016).

### Immunohistochemistry Analysis

Immunohistochemical examinations were performed to determine ADH1B levels in the hippocampus of model mice. Three mice per group were separated and primed with saline solution. Perfusion was then conducted with 4% paraformaldehyde. Brain tissues were removed and cut through the mid-sagittal plane. Brain hemispheres were fixed with 4% paraformaldehyde overnight and then embedded with paraffin. Paraffin sections were dried for 1 h (60°C) and dewaxed with xylene. After washing with a graded series of ethanol solutions, incubating with 3% H_2_O_2_, and blocking with 5% BSA, slides were incubated with primary antibody (anti-ADH1B, 1:100) overnight at 4°C, rinsed with PBS, and incubated with secondary antibody for 20 min at room temperature. Slides were visualized with diaminobenzidine. Mean integral optical density (IOD) was calculated in three fields of the hippocampus for each slide. Each field was imaged at 400× magnification using a microscope (Leica, DM3000) equipped with Image-Pro Plus 6.0 (Media Cybernetics, Rockville, MD, United States) (Lian et al., 2017).

### Cell Viability Assay

SH-SY5Y cells were seeded in 96-well plates 48 h after transfection and incubated with 0.1% DMSO in OPTI-MEM medium (Thermo Fisher Scientific, Waltham, MA, United States) or Aβ1-42 (10 μM) in OPTI-MEM medium for an additional 12 h. Cells were incubated with 10 μL of WST-8 for 3.5 h at 37°C. Absorbance values at 450 nm were determined using an iMARK microplate reader (Bio-Rad, Hercules, CA, United States).

### Analysis of Apoptosis

Apoptosis of SH-SY5Y cells induced by Aβ1-42 (10 μM) was determined using a Annexin V-FITC/PI apoptosis detection kit (Beijing 4A Biotech) according to the manufacturer’s instructions. Annexin V positive and PI negative cells were considered apoptotic. Apoptosis rates were determined using flow cytometry (BD, C6, United States). TUNEL apoptosis assays were also performed using a TUNEL Apoptosis Assay Kit (Beyotime, Beijing). Apoptotic cells were observed under a fluorescence microscope.

### Measurement of Intracellular ROS Induced by Aβ1-42

Intracellular ROS levels were measured using fluorescence microscopy with 2′,7′-dichlorofluorescein diacetate (DCF-DA). Pretreated SH-SY5Y cells were harvested from a flask and plated at 1 × 10^4^ cells per well on six-well plates. The 2′,7′-DCF-DA-stained SH-SY5Y cells were visualized using a Nikon ECLIPSE Ci fluorescence microscope as described previously (Chandrasekaran et al., 2012). Results are expressed as arbitrary units of fluorescence intensity per 10^4^ cells from at least three independent experiments per group. In addition, total superoxide dismutase (SOD) was detected using a Total SOD Assay kit (Beyotime, Beijing) according to the manufacturer’s instructions.

### Statistical Analysis

Statistical analysis was performed using GraphPad Prism software (version 5.01; California, United States). Data from each experimental condition were obtained from at least three independent experiments. Data are presented as mean ± S.D. Statistical significance was evaluated using one-way or two-way ANOVA or *t*-tests (as appropriate). Chi-square tests were used to compare groups of categorical data. Mann–Whitney *U*-tests were used to compare continuous data. Statistical significance was considered for *p* < 0.05.

## Results

### Lower Serum Levels of ADH1B in AD Patients

**FIGURE 1:**
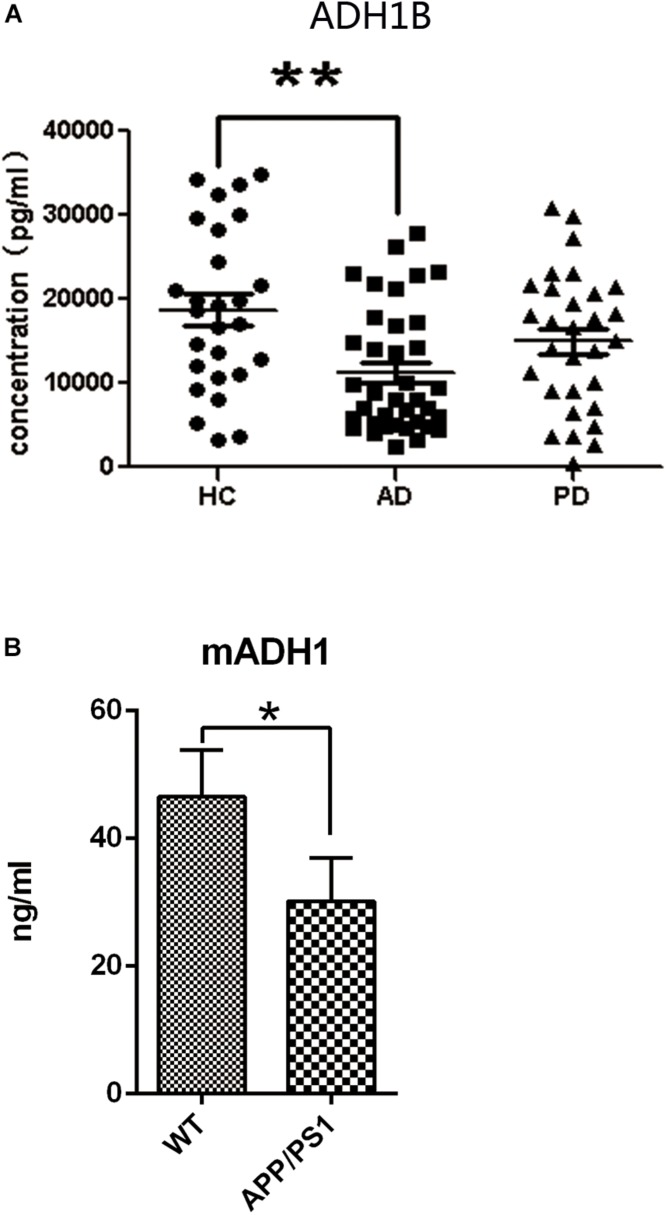
Serum expression levels of alcohol dehydrogenase 1B (ADH1B) in healthy controls (HC), Alzheimer’s disease (AD), and Parkinson’s disease (PD) patients and in AD model mice determined using enzyme-linked immunosorbent assays. **(A)** Analysis showed that ADH1B was down-regulated in AD patients (*n* = 37) compared to the HC group (*n* = 27, Mann–Whitney *U*-test, ^∗∗^*p* < 0.01). No significant differences were observed between PD (*n* = 30) and HC groups (*p* > 0.05). **(B)** Serum expression levels of ADH1 in AD model mice after 8 months (*n* = 5 per group, *t*-test). Data are shown as mean ± S.D. ^*^p < 0.05 and ^∗∗^*p* < 0.01.

Demographic characteristics of the enrolled populations are displayed in Table 1. No significant difference in age and gender between AD and PD patients were observed when compared with the health control (HC) group. Serum ADH1B levels were determined using ELISA. Interestingly, lower ADH1B levels were observed in AD patients than in the PD and HC groups (Figure 1, *p* < 0.01). PD and AD are both common neurodegenerative diseases (Fisher et al., 2015), and our data show that ADH1B levels in PD and HC were not significantly different (*p* > 0.05). These results suggest that ADH1B could be specifically related to AD.

### Age-Dependent Decrease in ADH1 Levels in the Hippocampus of APP/PS-1 AD Model Mice

**FIGURE 2:**
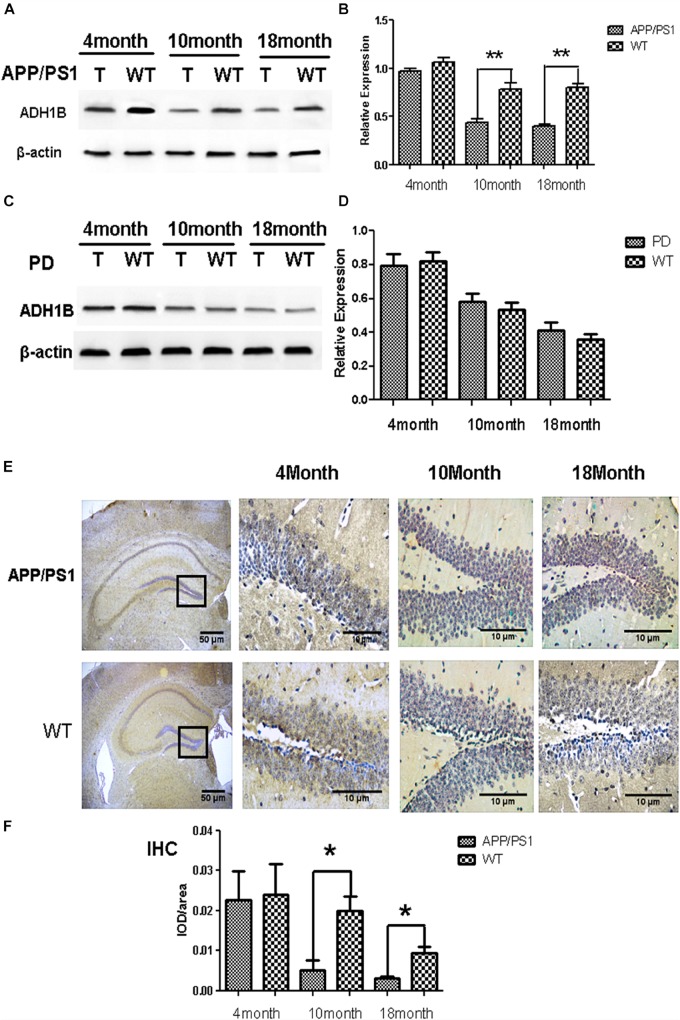
Alcohol dehydrogenase 1B (ADH1B) in the hippocampus of Alzheimer’s disease (AD) and Parkinson’s disease (PD) model mice at different ages. **(A)** Representative ADH1B polypeptides detected in the hippocampus of APP/PS-1 AD model mice with 4 (4T), 10 (10T), and 18 (18T) months of age. Negative controls with similar ages (4WT, 10WT, and 18WT) were used. Proteins were extracted from the hippocampus of model mice and then analyzed using western blotting. **(B)** Relative expression of ADH1B in the hippocampus of APP/PS-1 AD model mice (*n* = 8 per group, two-way ANOVA). **(C)** Representative signals of ADH1B in the hippocampus of Prnp-SNCA^*^A53T PD model mice. **(D)** Relative expression of ADH1B in the hippocampus of Prnp-SNCA^*^A53T PD model mice (*n* = 8 per group, two-way ANOVA). β-Actin was used as a loading control. **(E)** Immunohistochemical staining of paraffin sections of mouse brain tissue using anti-ADH1B antibody (*n* = 4 per group). Red arrows, ADH1B-positive regions. Scale bar, 50 μm. **(F)** Relative expression of ADH1B in the hippocampus determined using immunohistochemical staining (Chi-square tests). Data are shown as mean ± S.D. ^*^p < 0.05 and ^∗∗^*p* < 0.01.

Human ADH1 consists of three isoenzymes, ADH1A, ADH1B, and ADH1C. Rodents, however, do not show this diversity (Duester et al., 1999), and we therefore assessed only the expression of ADH1 in mice. We found that ADH1 levels in the serum of APP/PS1 model mice decreased significantly, similar to what was observed in human serum (Figure 1B). ADH1 levels in the hippocampus of APP/PS-1 AD model mice were determined using western blotting (Figures 2A,B). Our results show that the ADH1 protein levels dramatically declined with aging (Figures 2A–D). Furthermore, ADH1 levels in APP/PS-1 double transgenic mice declined compared with age-matched control mice at the ages of 10 (*p* < 0.01) and 18 (*p* < 0.01) months. However, no difference in ADH1 levels were observed in the PD transgenic mice model (Figures 2C,D, *p* > 0.05). These data indicate that ADH1 levels might be associated with AD, especially in some age groups. ADH1 levels were confirmed using immunochemistry (IHC) of paraffin sections of APP/PS-1 mouse brain tissue (Figure 2E). Expression of this protein in 10-month-old and 18-month-old mice were lower than that in aged-matched mice of the control group (Figure 2F, *p* < 0.05).

### Down-Regulation of ADH1B Induced by Aβ1-42 in SH-SY5Y Cell Line

**FIGURE 3:**
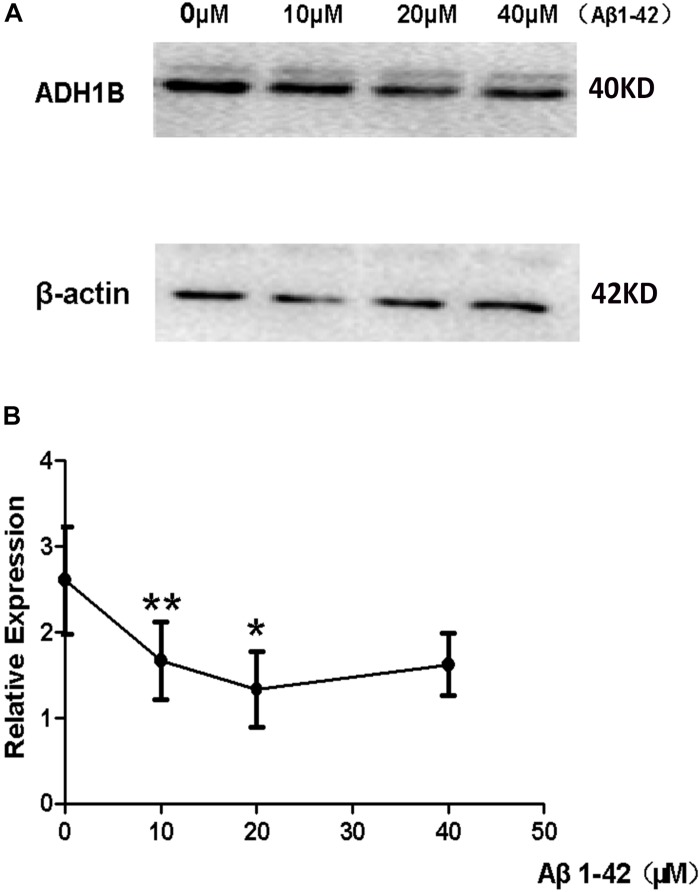
Effects of β-amyloid (Aβ)1-42 on alcohol dehydrogenase 1B (ADH1B) expression in SH-SY5Y cells. SH-SY5Y cells were pretreated with Aβ1-42 (10, 20, and 40 μM) or 0.1% DMSO. **(A)** Representative signals of ADH1B treated with different concentration of Aβ1-42 or 0.1% DMSO. **(B)** Relative expression of ADH1B. β-Actin was used as a loading control. Data are shown as mean ± S.D. of three separate experiments performed in triplicate. Data were compared with independent samples using one-way ANOVA. ^*^p < 0.05 and ^∗∗^*p* < 0.01.

The deposition of Aβ1-42 plays a very important role in the occurrence and development of AD (Fukuyama et al., 2000; Huang et al., 2012). Our previous results have demonstrated that ADH1B levels decrease in the serum of AD patients (see Figure 1) and the hippocampus of AD model mice (see Figure 2A). To investigate the effects of Aβ1-42 on ADH1B protein levels, we designed an experiment in which SH-SY5Y cells were treated with different concentrations of Aβ1-42 (10, 20, and 40 μM). The results show that the ADH1B protein levels in cells treated with 10 μM Aβ1-42 declined significantly (Figures 3A,B, *p* < 0.01). However, the reduction in ADH1B protein levels was not clear when increasing the dose of Aβ1-42 in SH-SY5Y cells. The data indicate that Aβ1-42 can down-regulate the expression of ADH1B especially in low concentrations.

### ADH1B-Mediated Reduction of Apoptotic Rate of SH-SY5Y Cells Cultured With Aβ1-42

**FIGURE 4:**
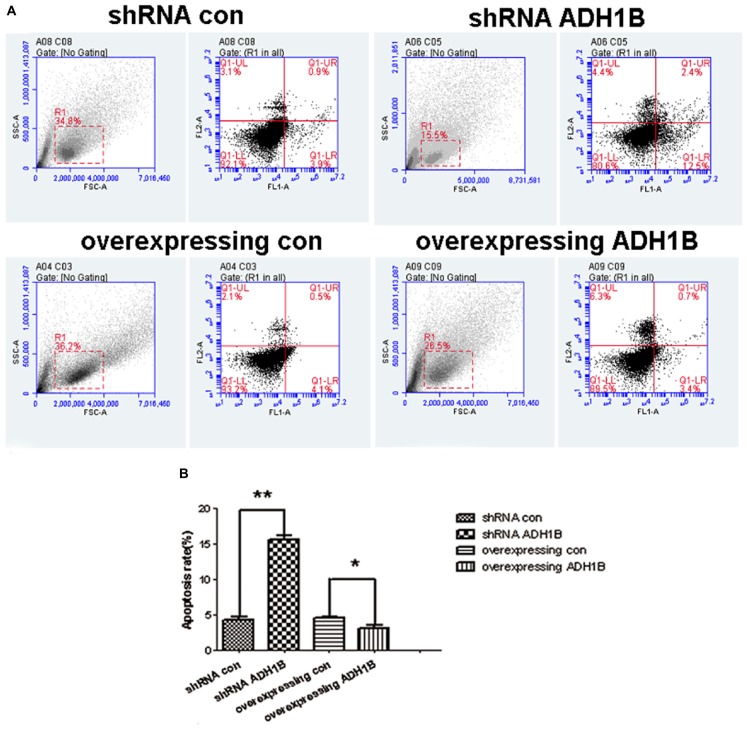
Effects of alcohol dehydrogenase 1B (ADH1B) on the apoptotic rate of SH-SY5Y cells. After treatment with 10 μM Aβ1-42, cells were divided into four groups: overexpressing ADH1B, overexpressing control, shRNA ADH1B, and shRNA control. **(A)** Flow cytometry (FCM) analysis of apoptosis of SH-SY5Y cells. Cell apoptosis was identified using Annexin-V/propidium iodide (PI) double-staining assays. Q2-1, Q2-2, Q2-3, and Q2-4 indicate viable (live), early apoptotic, late apoptotic, and necrotic regions, respectively. **(B)** Apoptotic rate of cells were determined using FCM. Data are shown as mean ± S.D. of three separate experiments performed in triplicate. Data were compared with independent samples using Chi-square tests. ^*^p < 0.05 and ^∗∗^*p* < 0.01.

The apoptotic rates of SH-SY5Y cells induced by Aβ1-42 were determined using flow cytometry (FCM) to confirm whether ADH1B could influence apoptosis. The results show that the apoptotic rate of cells transfected with the shRNA ADH1B vector and cultured with Aβ1-42 was more than 3-fold higher than that of control cells (35.51 vs. 10.66%, Figures 4A,B, *p* < 0.01). In contrast, the apoptotic rate of cells transfected with the ADH1B-overexpression vector decreased slightly compared with control cells (5.49 vs. 11.03%), but it could be significant considering the non-reproducibility of neurons (Figures 4A,B). In addition, TUNEL staining assays were also performed to determine apoptotic cells (Supplementary Figure 1). Fluorescence intensity of positive apoptotic cells was the strongest after transfection with the shRNA ADH1B vector, whereas positive apoptotic cells were clearly reduced after transfection with the ADH1B-overexpression vector. These results indicate that ADH1B could reduce apoptosis of SH-SY5Y cells stimulated with Aβ1-42.

### ADH1B-Mediated Promotion of SH-SY5Y Cell Viability

**FIGURE 5:**
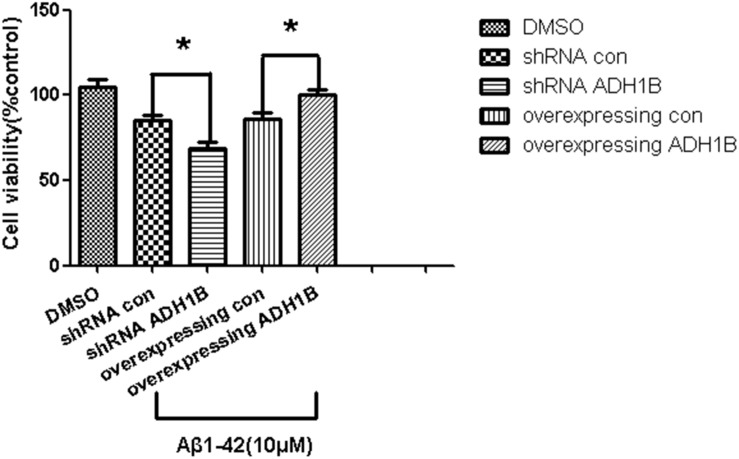
Effects of alcohol dehydrogenase 1B (ADH1B) on the viability of SH-SY5Y cells cultured with β-amyloid (Aβ)1-42. Viability of cells transfected with shRNA vector or ADH1B overexpression vector and cultured with 0.1% DMSO or 10 μM Aβ1-42 for 12 h was determined using MTT assays. Data are shown as mean ± S.D. of three separate experiments performed in triplicate. The data were compared with the independent samples using One-way ANOVA.^*^p < 0.05.

The effects of ADH1B on the viability of SH-SY5Y cells was determined with the MTT assay. The data show that the viability of cells transfected with the shRNA ADH1B vector and cultured with 10 μM Aβ1-42 decreased significantly compared with control cells. In addition, the viability of cells transfected with the ADH1B-overexpression vector significantly increased compared with the control (*p* < 0.05). These results indicate that ADH1B could protect SH-SY5Y cells against the toxicity induced by Aβ1-42 (Figure 5).

### Down-Regulation of p75NTR by ADH1B

**FIGURE 6:**
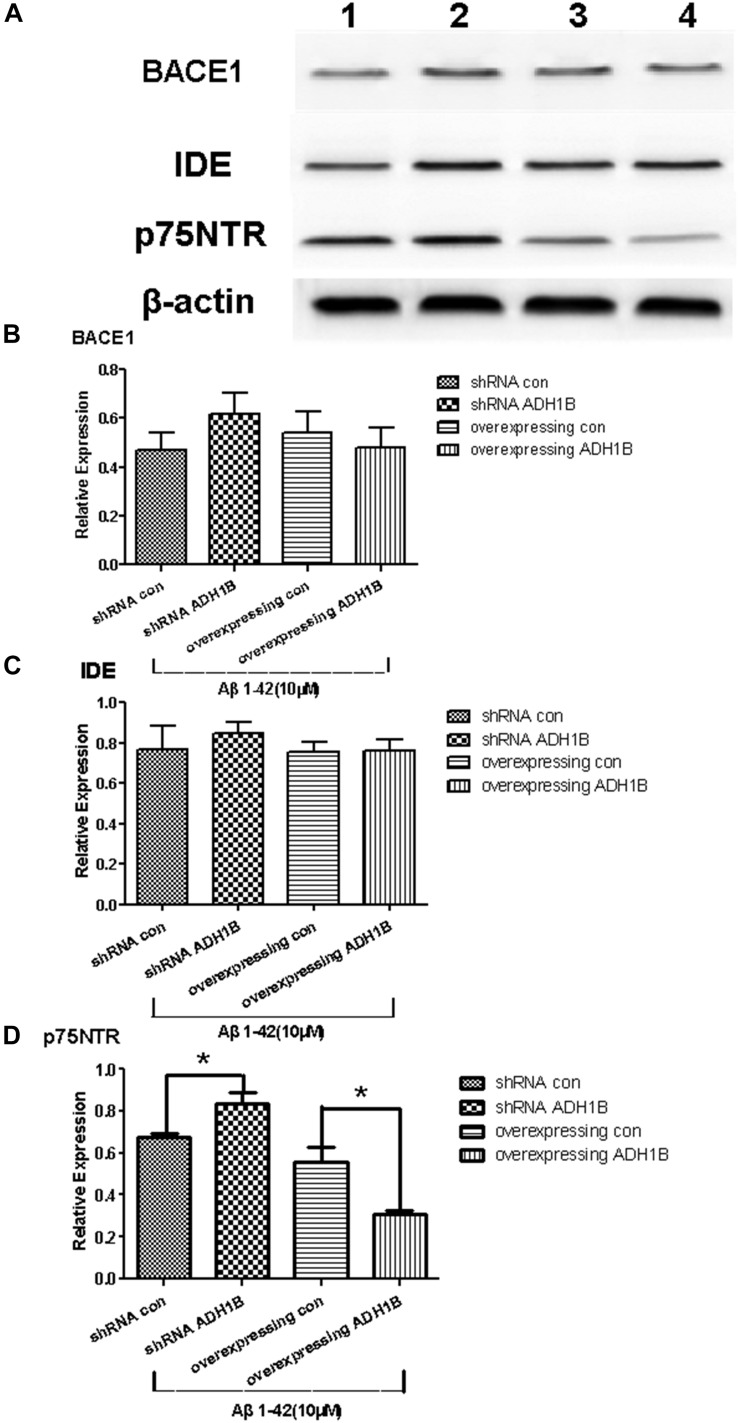
Effects of alcohol dehydrogenase 1B (ADH1B) on BACE 1, IDE, and p75NTR expression in SH-SY5Y cells cultured with β-amyloid (Aβ)1-42. After treatment with 10 μM Aβ1-42, cells were divided into four groups: overexpressing ADH1B, overexpressing control, shRNA ADH1B, and shRNA control. **(A)** Representative expression of Aβ42-related proteins in different groups was determined. Proteins were analyzed using western blotting with antibodies against BACE 1, IDE, and p75NTR. Lane 1, shRNA con group; lane 2, shRNA ADH1B group; lane 3, overexpressing con group; lane 4, overexpressing ADH1B group. **(B)** Relative quantitation of BACE 1 in the four groups. **(C)** Relative quantitation of IDE in the four groups. **(D)** Relative quantitation of p75NTR in the four groups. β-Actin was used as a loading control. Data are shown as mean ± S.D. of three separate experiments performed in triplicate. The data were compared with the independent samples using One-way ANOVA.^*^p < 0.05.

It is reported that p75NTR is an Aβ receptor that mediates Aβ-induced neurodegenerative signals (Jiao et al., 2015; Yao et al., 2015). BACE 1 is a rate-limiting enzyme in the cleavage of the APP to Aβ peptides (Durairajan et al., 2017), and IDE is a major endogenous Aβ-degrading enzyme. The levels and enzymatic activity of IDE are negatively correlated with the size of the amyloid plaques and AD pathology (Matioli and Nitrini, 2015). To investigate the effects of ADH1B on these proteins (p75NTR, BACE 1, and IDE) related to Aβ1-42 metabolism, we established ADH1B-overexpression and ADH1B-shRNA lentiviral vectors. These vectors were then transfected into SH-SY5Y cells treated with 10 μM Aβ1-42 (Arai et al., 2016). The empty lentiviral vector was used as negative control. The results show that the expression of p75NTR significantly increased and decreased in the shRNA ADH1B and ADH1B-overexpressing groups, respectively, compared with the corresponding controls (Figure 6D, *p* < 0.05). In contrast, no difference was observed in BACE 1 or IDE protein levels between these two transfected groups and the control group (Figures 6A–C, *p* > 0.05). These results strongly suggest that ADH1B attenuates Aβ-induced neurodegenerative signals by decreasing p75NTR levels.

### Regulation of Apoptosis-Related Protein Expression by ADH1B

**FIGURE 7:**
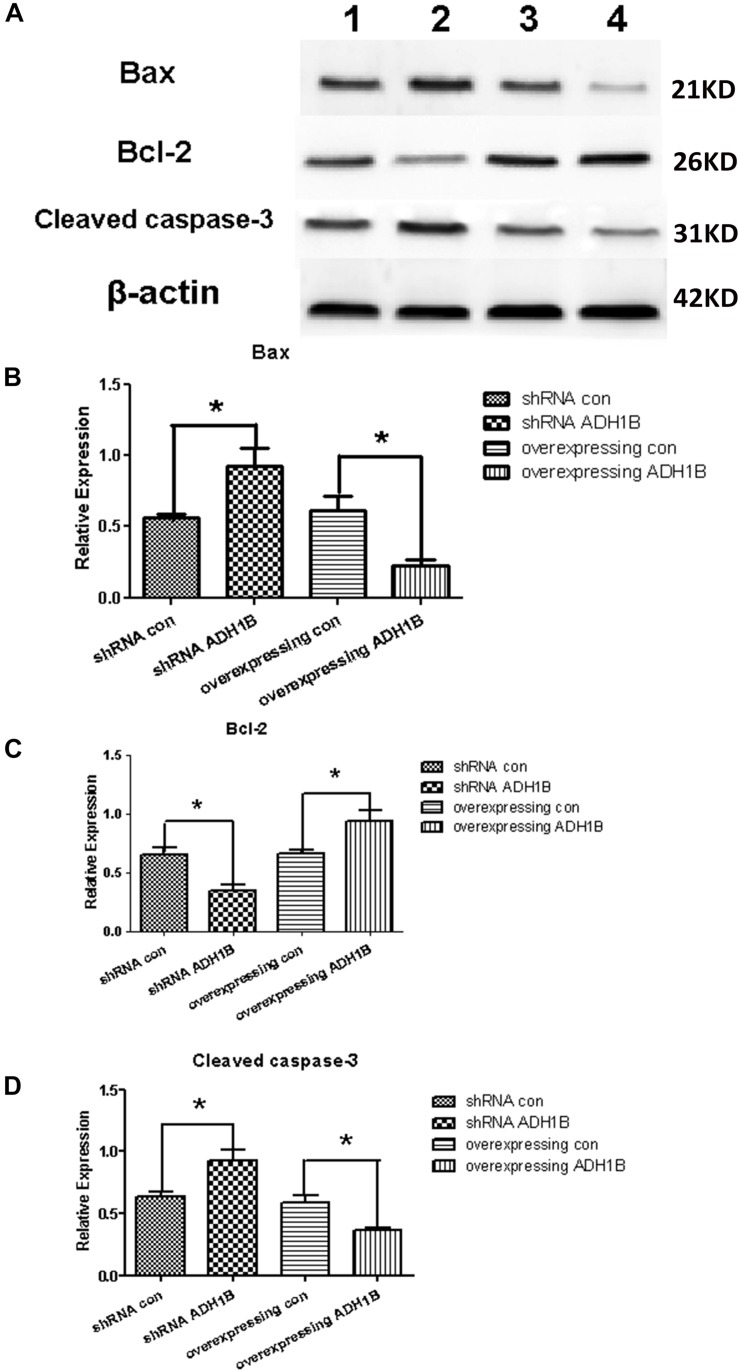
Effects of alcohol dehydrogenase 1B (ADH1B) on the expression of apoptosis-related proteins in SH-SY5Y cells. Protein levels of Bax, Bcl-2, and cleaved caspase-3 were measured using western blotting in cells transfected with lentivirus vectors and cultured with 10 μM β-amyloid (Aβ)1-42 for 12 h. **(A)** Representative expression of apoptosis-related proteins in different groups were detected. Proteins were analyzed using western blotting with antibodies against cleaved caspase-3, Bax, and Bcl-2. Lane 1, shRNA con group; lane 2, shRNA ADH1B group; lane 3, overexpressing con group; lane 4, overexpressing ADH1B group. **(B)** Relative quantitation of Bax in the four groups. **(C)** Relative quantitation of Bcl-2 in the four groups. **(D)** Relative quantitation of cleaved caspase-3 in the four groups. β-Actin was used as a loading control. Data are shown as mean ± S.D. of three separate experiments performed in triplicate. The data were compared with the independent samples using One-way ANOVA. ^*^p < 0.05.

Cleaved caspase-3 and Bax have been demonstrated to promote apoptosis (Bressenot et al., 2009; Kirkland et al., 2010), whereas Bcl-2 reduces the apoptotic rate (Maranci et al., 2010). To uncover the mechanisms by which ADH1B down-regulates apoptosis, the levels of apoptosis-related proteins were determined using western blotting. The results show that the protein levels of cleaved caspase-3 and Bax increased significantly in cells transfected with the shRNA ADH1B vector, whereas the corresponding protein levels of cells transfected with the ADH1B-overexpression vector decreased significantly compared with the control (Figures 7A,B,D, *p* < 0.05). In contrast, Bcl-2 levels significantly decreased and increased in cells transfected with the shRNA ADH1B and ADH1B-overexpression vectors, respectively, compared with control cells (Figures 7A,C, *p* < 0.05). These results show that ADH1B plays a very important role in regulating the levels of apoptosis-related proteins.

### ADH1B-Mediated Protection of SH-SY5Y Cells Against Oxidative Stress Injury Induced by Aβ1-42

**FIGURE 8:**
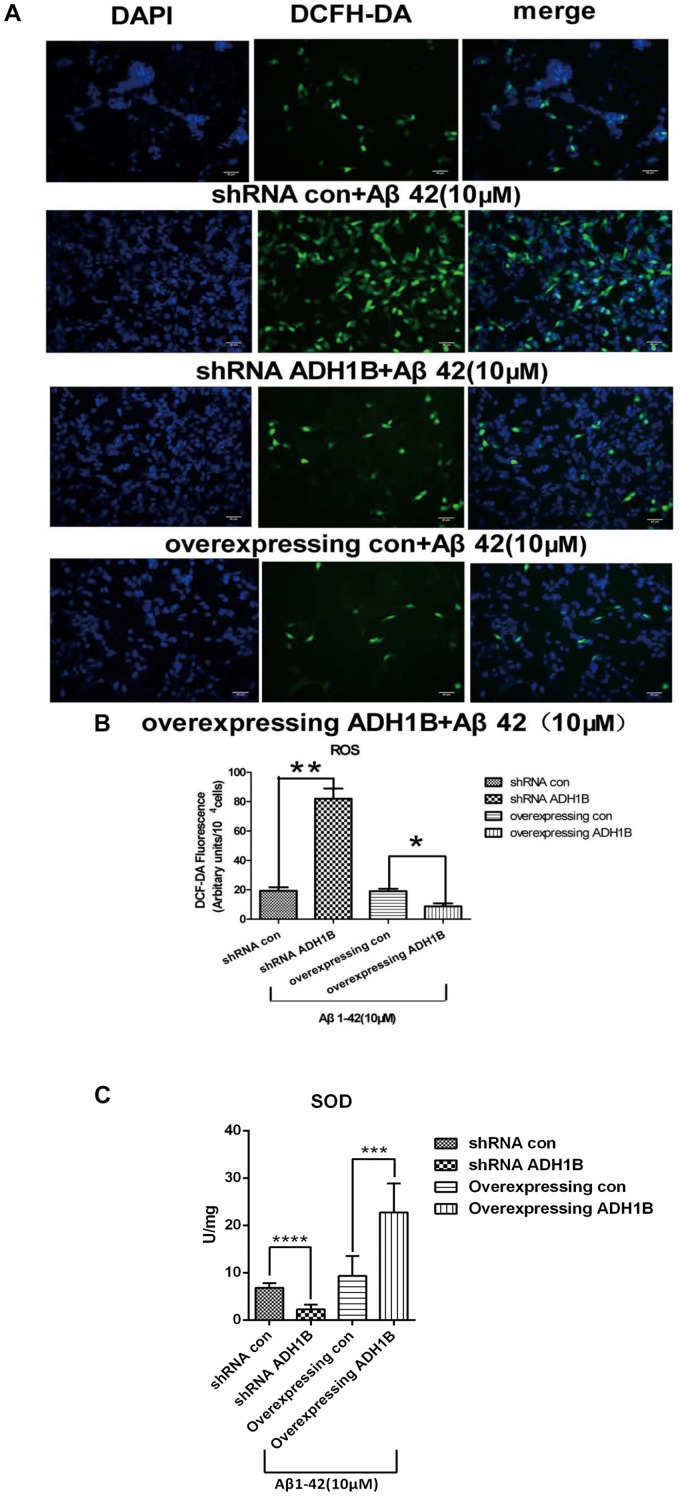
Effects of alcohol dehydrogenase 1B (ADH1B) on the levels of reactive oxygen species (ROS) products in SH-SY5Y cells cultured with β-amyloid (Aβ)1-42. Cells transfected with shRNA or ADH1B overexpression vectors and cultured with 10 μM Aβ1-42 for 12 h were analyzed using fluorescence spectrophotometry. **(A)** Photomicrographs of 2′,7′-DCF-DA-stained SH-SY5Y cells visualized with a fluorescence microscope. 4′,6′-Diamidino-2-phenylindole (DAPI), blue color; DCF-DA, green color. Scale bar, 40 μm. **(B)** Intracellular ROS levels were measured using fluorescence spectrophotometry and 2′,7′-DCF-DA as a probe. Results are expressed as arbitrary units of fluorescence intensity per 10^4^ cells from at least three independent experiments per group. Data were compared with independent samples using Chi-square tests. **(C)** SOD activity in SH-SY5Y cells. Values are shown as mean ± S.D. Data were compared with independent samples using one-way ANOVA.^*^p < 0.05, ^∗∗^*p* < 0.01, ^∗∗∗^*p* < 0.001, and ^****^p < 0.0001.

Previous studies have found that Aβ1-42 can induce oxidized stress damage and cell apoptosis at the 10–40 μM concentration range (Arai et al., 2016). To investigate the effects of ADH1B on the oxidative stress levels, intracellular ROS levels were determined using fluorescence microscopy with 2′,7′-dichlorofluorescein diacetate (DCF-DA). Following treatment of cells with 10 μM Aβ1-42 for 12 h, a 4.2-fold increase in intracellular ROS generation was observed in the shRNA ADH1B group compared with the control group (*p* < 0.01). In contrast, overexpression of ADH1B resulted in antioxidative activity in SH-SY5Y cell lines (Figures 8A,B, *p* < 0.05). SOD is an important antioxidative enzyme. Our results show that SOD activity in cells overexpressing ADH1B was 2.5-fold higher than that of the control group (*p* < 0.001), whereas SOD activity in the shRNA ADH1B group decreased significantly (Figure 8C, *p* < 0.0001). These results suggest that ADH1B reduces intracellular ROS levels and protects the cell against oxidative stress damage induced by Aβ1-42.

## Discussion

A recent large-scale study has demonstrated that frequent alcohol consumption is associated with elevated risk of dementia (Langballe et al., 2015). Ethanol is not toxic to the human body, whereas acetaldehyde from oxidized ethanol is toxic. Acetaldehyde, the substrate of ADH and ALDH, mediates cognitive impairment and brain damage (Hernandez-Collados et al., 1997). ALDH decreases 4-HNE levels and could be involved in AD pathology (Benedetti et al., 2014). In addition, ADH1B polymorphism was found to be associated with the risk of AD. Ma et al. reported that the AA genotype of ADH1B rs1229984 is associated with an increased risk of AD (Ma and Lu, 2016). However, its exact molecular mechanism related to AD remains unknown. In the present study, we compared the ADH1B levels in the serum of AD patients, PD patients, and HCs and found that ADH1B is reduced only in the serum of AD patients but not of PD patients. Although AD and PD are all considered neurodegenerative diseases, PD is characterized by loss of dopamine (DA) neurons in substantia nigra pars compacta (SNPC) and later in the ventral tegmental area. These events are accompanied by progressive loss of DA innervations of the nucleus caudatus and putamen, resulting primarily in movement disabilities. A previous study showed that genetic variants of the G78stop mutation in ADH1C and related ADH4 SNPs are associated with PD (Buervenich et al., 2005), suggesting that other ADHs but not ADH1B might participated in PD. Moreover, our results show that ADH1 levels in the serum and hippocampus of APP/PS-1 AD model mice decreased significantly (Figures 1, 2). The deposition of Aβ1-42, a degradation product of APP cleaved by BACE-1 and the γ-secretase complex, plays a very important role in AD pathology (Jamsa et al., 2011). Neurons incubated with Aβ1-42 are currently widely used in the study of AD (Han et al., 2017; Zhang et al., 2017). Here, we used SH-SY5Y cells cultured with Aβ1-42 as an AD cell model. The down-regulation of ADH1B induced by Aβ1-42 was also observed in the cell model (Figure 3). These results indicate that ADH1B may be associated with AD pathology.

Recent evidence has confirmed that Aβ-induced neurotoxicity results in cell apoptosis (Du et al., 2019; Wang et al., 2019). Thus, the effects of ADH1B on the apoptosis rate and viability of AD model cells were first evaluated. The apoptotic rate was elevated 3-fold after shRNA interference of ADH1B, and reduced after overexpression of ADH1B (Figure 4). Moreover, the viability of the AD model cells was found to be elevated by ADH1B, which coincided with its down-regulation of apoptosis. These results show that ADH1B suppresses the apoptosis of the AD model cells.

p75NTR, an Aβ receptor, mediates Aβ-induced neurodegenerative signals and promotes apoptosis induced by pro-neurotrophins (Jiao et al., 2015; Yao et al., 2015). BACE-1 and IDE play key roles in the production and degradation, respectively, of Aβ1-42 (Matioli and Nitrini, 2015; Durairajan et al., 2017). Our results show that p75NTR was down-regulated by ADH1B (Figure 6D). In contrast, no effect of ADH1B was observed on the expression of BACE-1 and IDE. These results suggest that ADH1B attenuates Aβ1-42-induced neurodegenerative signals by decreasing p75NTR levels, which could be one of the key mechanisms of ADH1B reducing AD cell apoptosis.

Several studies demonstrated that cleaved caspase-3 and Bax promote apoptosis through poly ADP-ribose polymerase (PARP) and mitochondrial stress, respectively (Wang et al., 2006; Dingeldein et al., 2017). In addition, Bcl-2 reduces the apoptotic rate (Maranci et al., 2010). In the present study, cleaved caspase-3 and Bax were found to be down-regulated after overexpression of ADH1B, and up-regulated after shRNA interference of ADH1B. In contrast, Bcl-2 levels were up-regulated in AD model cells transfected with the ADH1B overexpression vector. These results strongly indicate that ADH1B decreases apoptosis through pathways associated with cleaved caspase-3, Bax, and Bcl-2 in AD model cells.

Previous studies have indicated that Aβ can bring about oxidative injury to neurons (Xu et al., 2016; Du et al., 2019). Oxidative damage of macromolecules in neurons may participate in the pathogenesis of AD (Keller et al., 2005). APP levels are raised, and the vicious pathophysiological cycles initiated by tau phosphorylation result in high levels of oxidative stress (Gibson, 2002). Our results demonstrate that ADH1B clearly reduces ROS levels in AD model cells, suggesting that ADH1B protects SH-SY5Y cells against oxidative stress injury induced by Aβ1-42. In summary, our results show that ADH1B suppresses apoptosis through attenuating Aβ1-42-induced neurodegenerative signals mediated by p75NTR, regulating the expression of apoptosis-related proteins and reducing oxidative stress levels in SH-SY5Y cells. These results also indicate that ADH1B might be important in the prevention of AD, especially for alcohol abusers, and might be a potential new target of AD treatment.

## Ethics Statement

This study was carried out in accordance with the recommendations of ethics committee of Xuanwu Hospital of Capital Medical University with written informed consent from all subjects. All subjects gave written informed consent in accordance with the Declaration of Helsinki. The protocol was approved by the ethics committee of Xuanwu Hospital of Capital Medical University. All animal experiments conformed to the National Institutes of Health guidelines. All animal procedures were approved by the ethics committee of Xuanwu Hospital of Capital Medical University.

## Author Contributions

YW, YZ, and PW designed the experiments and wrote the manuscript. YW, YZ, and XZ performed the experiments. CL and TY helped to collect serum samples. All authors reviewed and approved the final manuscript.

## Conflict of Interest Statement

The authors declare that the research was conducted in the absence of any commercial or financial relationships that could be construed as a potential conflict of interest.

## Abbreviations

4-HNE: 4-hydroxy-2-nonenal
Aβ: β-amyloid
AD: Alzheimer’s disease
ADH1B: alcohol dehydrogenase 1B
ALDH2: mitochondrial aldehyde dehydrogenase
BACE 1: β-site amyloid precursor protein cleaving enzyme 1
HC: healthy control
IDE: insulin-degrading enzyme
IHC: immunohistochemistry
MDA: malondialdehyde
p75NTR: p75 neurotrophin receptor
PD: Parkinson’s disease
ROS: reactive oxygen species.
