# Supplementary material for: Alcohol Dehydrogenase 1B Suppresses β-Amyloid-Induced Neuron Apoptosis
Source: Front Aging Neurosci. 2019 Jun 5;11:135. doi: 10.3389/fnagi.2019.00135 (PMC6560161; doi:10.3389/fnagi.2019.00135)
Supplement: Supplementary file 1 [file Data_Sheet_1.docx]

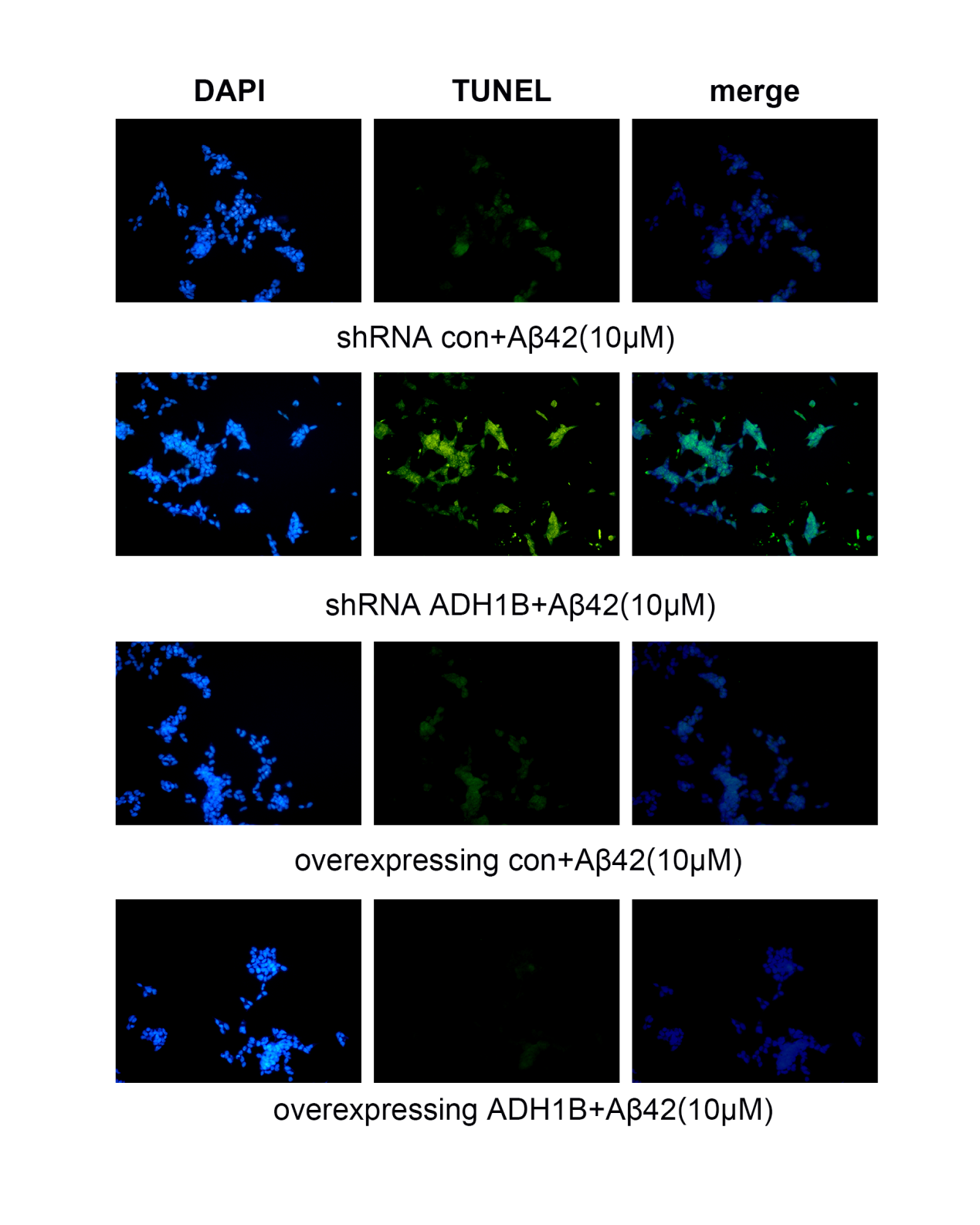


**Supplementary Figure 1.** TUNEL apoptosis staining assay of SH-SY5Y cells by fluorescence spectrophotometry (4',6'-diamidino-2-phenylindole (DAPI), blue color; TUNEL stained cells, green color; original magnification, ×200). Three separate experiments are performed in triplicate.
